# Ascorbate content of clinical glioma tissues is related to tumour grade and to global levels of 5-hydroxymethyl cytosine

**DOI:** 10.1038/s41598-022-19032-8

**Published:** 2022-09-01

**Authors:** Rebekah L. I. Crake, Eleanor R. Burgess, George A. R. Wiggins, Nicholas J. Magon, Andrew B. Das, Margreet C. M. Vissers, Helen R. Morrin, Janice A. Royds, Tania L. Slatter, Bridget A. Robinson, Elisabeth Phillips, Gabi U. Dachs

**Affiliations:** 1 Mackenzie Cancer Research Group, Department of Pathology and Biomedical Science, University of Otago Christchurch, Christchurch, New Zealand; 2 Metastasis Research Laboratory, GIGA-Cancer, University of Liège, Liege, Belgium; 3 Centre for Free Radical Research, Department of Pathology and Biomedical Science, University of Otago Christchurch, Christchurch, New Zealand; 4 Peter MacCallum Cancer Centre, 305 Grattan St, Melbourne, VIC Australia; 5 Sir Peter MacCallum Department of Oncology, The University of Melbourne, Parkville, VIC Australia; 6 Cancer Society Tissue Bank, University of Otago Christchurch, Christchurch, New Zealand; 7 Department of Pathology, Dunedin School of Medicine, University of Otago, Dunedin, New Zealand; 8 Canterbury Regional Cancer and Haematology Service, Canterbury District Health Board, Christchurch, New Zealand; 9 Department of Medicine, University of Otago Christchurch, Christchurch, New Zealand

**Keywords:** Cancer microenvironment, CNS cancer, Cancer, Oncology

## Abstract

Gliomas are incurable brain cancers with poor prognosis, with epigenetic dysregulation being a distinctive feature. 5-hydroxymethylcytosine (5-hmC), an intermediate generated in the demethylation of 5-methylcytosine, is present at reduced levels in glioma tissue compared with normal brain, and that higher levels of 5-hmC are associated with improved patient survival. DNA demethylation is enzymatically driven by the ten–eleven translocation (TET) dioxygenases that require ascorbate as an essential cofactor. There is limited data on ascorbate in gliomas and the relationship between ascorbate and 5-hmC in gliomas has never been reported. Clinical glioma samples (11 low-grade, 26 high-grade) were analysed for ascorbate, global DNA methylation and hydroxymethylation, and methylation status of the O-6-methylguanine-DNA methyltransferase (MGMT) promoter. Low-grade gliomas contained significantly higher levels of ascorbate than high-grade gliomas (*p* = 0.026). Levels of 5-hmC were significantly higher in low-grade than high-grade glioma (*p* = 0.0013). There was a strong association between higher ascorbate and higher 5-hmC (*p* = 0.004). Gliomas with unmethylated and methylated MGMT promoters had similar ascorbate levels (*p* = 0.96). One mechanism by which epigenetic modifications could occur is through ascorbate-mediated optimisation of TET activity in gliomas. These findings open the door to clinical intervention trials in patients with glioma to provide both mechanistic information and potential avenues for adjuvant ascorbate therapy.

## Introduction

Gliomas are brain cancers that are incurable with a highly unfavourable prognosis; the median survival for high-grade glioma (HGG, grade IV, glioblastoma) with multimodal treatment is 11–15 months^1,2^. Low-grade gliomas (LGG, grades I–III) have significantly longer survival, but remain a terminal disease^1,2^. Current multimodal treatment for high-grade gliomas involves surgery with maximal possible resection, followed by radiotherapy with concomitant or adjuvant chemotherapy (oral temozolomide)^3^. Despite this, glioma recurrence is near universal^4,5^ and further treatment options are limited to a repeat of first-line treatment.

Glioma classification is moving from histopathology to genomics, transcriptomics and epigenomics classifiers^5–8^, with isocitrate dehydrogenase (*IDH1/2*) mutations and a chromosome 1p and 19q codeletion, among others, now commonly used for diagnosis and prognosis^7^. The presence of *IDH1/2* mutations is associated with a better prognosis whereas *IDH1/2* wild-type is associated with poorer prognosis^5^. Recently, the European Association of Neuro-Oncology have recommended the use of ‘astrocytoma grade 4’ instead of ‘*IDH* mutant glioblastoma’^6^; in our study we will use the term HGG to describe both IDH wild-type and IDH mutant tumours (glioblastoma, WHO grade IV).

Gliomas are characterised by aberrant DNA methylation, an epigenetic process that controls gene expression on a global scale without changes to the genetic code itself^1,9^. DNA methylation is a dynamic process that involves the generation of 5-methylcytosine (5-mC) by DNA methyltransferases (DNMTs). DNA methylation typically occurs on cytosines adjacent to a guanine, termed CpG, and heavily methylated CpG-rich regions, or ‘CpG islands’, are associated with gene silencing^1^. Demethylation occurs via the ten-eleven translocase enzymes (TET-1, -2, -3), which are dioxygenases that catalyse the oxidative conversion of 5-mC to 5-hydroxymethylcytosine (5-hmC)^10–12^.

Early genome-wide 5-mC analyses revealed that many LGGs and IDH-mutant HGG contain large numbers of hypermethylated loci, which is referred to as the glioma CpG island methylator phenotype (G-CIMP) and is closely associated with better prognosis^13–15^. In comparison, IDH-wild type HGG (glioblastoma) tend to have fewer hypermethylated loci, and worse prognosis^15^.

Compared to global methylation patterns, specific CpG islands in gene promoter regions are of particular interest. In glioma, methylation of the O-6-methylguanine-DNA methyltransferase (MGMT) promoter indicates an improved patient treatment response and, therefore, better prognosis^16–19^. MGMT is a DNA repair enzyme that is able to reverse the effects of alkylating chemotherapies such as temozolomide, and it is not expressed when the MGMT promoter region is hypermethylated^17^. Hypermethylation of the MGMT promoter is evident in 40–45% of gliomas^20,21^, with higher levels in LGG compared to HGG^22^. An association between MGMT promoter hypermethylation and presence of G-CIMP has been reported^23^.

In glioma, compared to normal brain tissue, TET2 shows reduced gene and protein expression^24,25^. *TET2* expression also decreases with increasing brain tumour grade, and lower *TET2* expression has been associated with worse overall survival^24^. Although the TET2 isoform is the most researched isoform, comparable findings have been reported for the other two isoforms^10,22^.

TETs belong to the 2-oxoglutarate dependent dioxygenase (2-OGDD) superfamily of enzymes, requiring 2-oxoglutarate (2-OG) and molecular oxygen as substrates, and ferrous iron (Fe^2+^) and ascorbate as co-factors (reviewed in^26^). Ascorbate is proposed to promote TET activity by maintaining iron in the Fe^2+^ state, and numerous studies have demonstrated increased TET activity in vitro and in vivo in response to ascorbate supplementation^27–30^. However, the relationship between ascorbate and 5-hmC in gliomas has never been reported, as measurements of ascorbate in brain cancer is limited to two small studies^31,32^. Landolt reported lower ascorbate levels in astrocytomas compared to normal brain (n = 11), and Burgess showed a relationship between ascorbate content of glioblastoma and patient’s survival (n = 37)^31,32^.

As a consequence of TET demethylases’ requirement for ascorbate, this study aimed to determine whether ascorbate levels in clinical glioma samples are associated with global levels of 5-hmC. We also investigated MGMT promoter hypermethylation status in the samples, as this is independently linked to prognosis. In this study, we have demonstrated that higher tumour ascorbate levels are associated with lower grade and elevated 5-hmC levels in clinical glioma samples. MGMT promoter methylation status was not associated with ascorbate content. Therefore, we propose that increasing tumour ascorbate content could shift global methylation levels in favour of improved glioma prognosis.

## Results

### Patient cohort and tumour pathology

The cohort consisted of 37 patients, 11 were diagnosed with low-grade glioma (grade I–III) and 26 with glioblastoma (Table 1). Most were male, NZ European, with an average age of 60 years (Table 1). All patients with LGG, which included ganglioglioma, ependymoma, astrocytoma and oligodendroglioma, were younger than 60 years (LGG mean age 39 years, HGG mean age 63 years). Following debulking surgery (with tissue collection), most patients had received chemotherapy and/or radiation (Table 1), with 54% of patients with HGGs receiving combined chemoradiation (60 Gy with temozolomide).

**Table 1:** Patient characteristics.

Characteristic	Number (%)	Grade I-III	Grade IV
	37 (100)	11 (100)	26 (100)
**Gender**			
Female	11 (30)	3 (27)	8 (31)
Male	26 (70)	8 (73)	18 (69)
**Age**			
≤ 60 years	19 (51)	11 (100)	8 (31)
> 60	18 (49)		18 (69)
**Ethnicity**			
Māori/Pacifica	2 (5)	0	2 (8)
NZ European	29 (78)	9 (82)	20 (77)
Other	5 (14)	1 (9)	4 (15)
Not declared	1 (3)	1 (9)	
**Diagnosis**			
Ganglioglioma	1 (3)	1 (9)	
Ependymoma	2 (5)	2 (18)	
Astrocytoma	4 (11)	4 (36)	
Oligodendroglioma	4 (11)	4 (36)	
Glioblastoma multiforme	26 (70)		26 (100)
**Post-surgery treatment**			
Radiation only	10 (27)	2 (18)	8 (31)
Chemotherapy only	2 (5)	2 (18)	
Chemoradiation	17 (46)	3 (27)	14 (54)
None	7 (19)	3 (27)	4 (15)
Unknown	1 (3)	1 (9)	

Tumours were located throughout the brain, with most located in the frontal or temporal lobes regardless of grade (Table 2). All HGG contained areas of necrosis (as expected), whereas most LGG did not (100% vs. 27% positive, *p* < 0.0001). Similarly, most HGG were positive for microvascular proliferation, whereas most LGG were negative (92% vs. 9% positive, *p* < 0.0001). According to MRI imaging, the median tumour diameter for all glioma tumours was estimated at 40 mm, with no difference between HGG and LGG. More than half of LGG tumours were *IDH1 R132H* mutants, but only two HGG were *IDH1 R132H* mutants (Table 2).

**Table 2:** Clinicopathological and molecular characterisation of glioma tumour samples.

Characteristic	Number (%)	Grade I-III	Grade IV	p*
	37 (100)	11 (100)	26 (100)	
**Position**				
Frontal	11 (30)	2 (18)	9 (35)	0.073
Parietal	8 (22)	1 (9)	7 (27)	
Temporal	14 (38)	6 (55)	8 (31)	
Occipital	2 (5)	0	2 (8)	
Cerebellar	2 (5)	2 (18)	0	
**Necrosis**				
Positive	29 (78)	3 (27)	26 (100)	** < 0.0001**
Negative	7 (19)	7 (64)	0	
Not recorded	1 (3)	1 (9)	0	
**Microvascular proliferation**				
Positive	25 (67)	1 (9)	24 (92)	** < 0.0001**
Negative	11 (30)	9 (82)	2 (8)	
Not recorded	1 (3)	1 (9)	0	
**Tumour size (on imaging)**				
≤ 40 mm	14 (38)	3 (27)	11 (42)	0.706
> 40 mm	22 (59)	7 (64)	15 (58)	
Not recorded	1 (3)	1 (9)	0	
***IDH1* status**				
Mutant R132H	8 (22)	6 (55)	2 (8)	**0.004**
Wild type	29 (78)	5 (45)	24 (92)	
**Ascorbate content**				
≤ median (0.338 nmol/μg DNA)	19 (51)	4 (36)	15 (58)	0.295
> median (0.338 nmol/μg DNA)	18 (49)	7 (64)	11 (42)	
**Global 5-hmC DNA status**				
≤ median (0.18% total C)	18 (49)	2 (18)	16 (62)	**0.0275**
> median (0.18% total C)	18 (49)	9 (82)	9 (35)	
Not determined	1 (2)		1 (3)	
**MGMT promoter status**				
methylated	12 (32)	4 (36)	8 (31)	> 0.99
unmethylated	25 (68)	7 (64)	18 (69)	

*Comparison of grade I–III versus grade IV, Chi-square or Fisher’s exact test, * bold indicates significant p values.

### Ascorbate content of glioma samples

**Figure 1:**
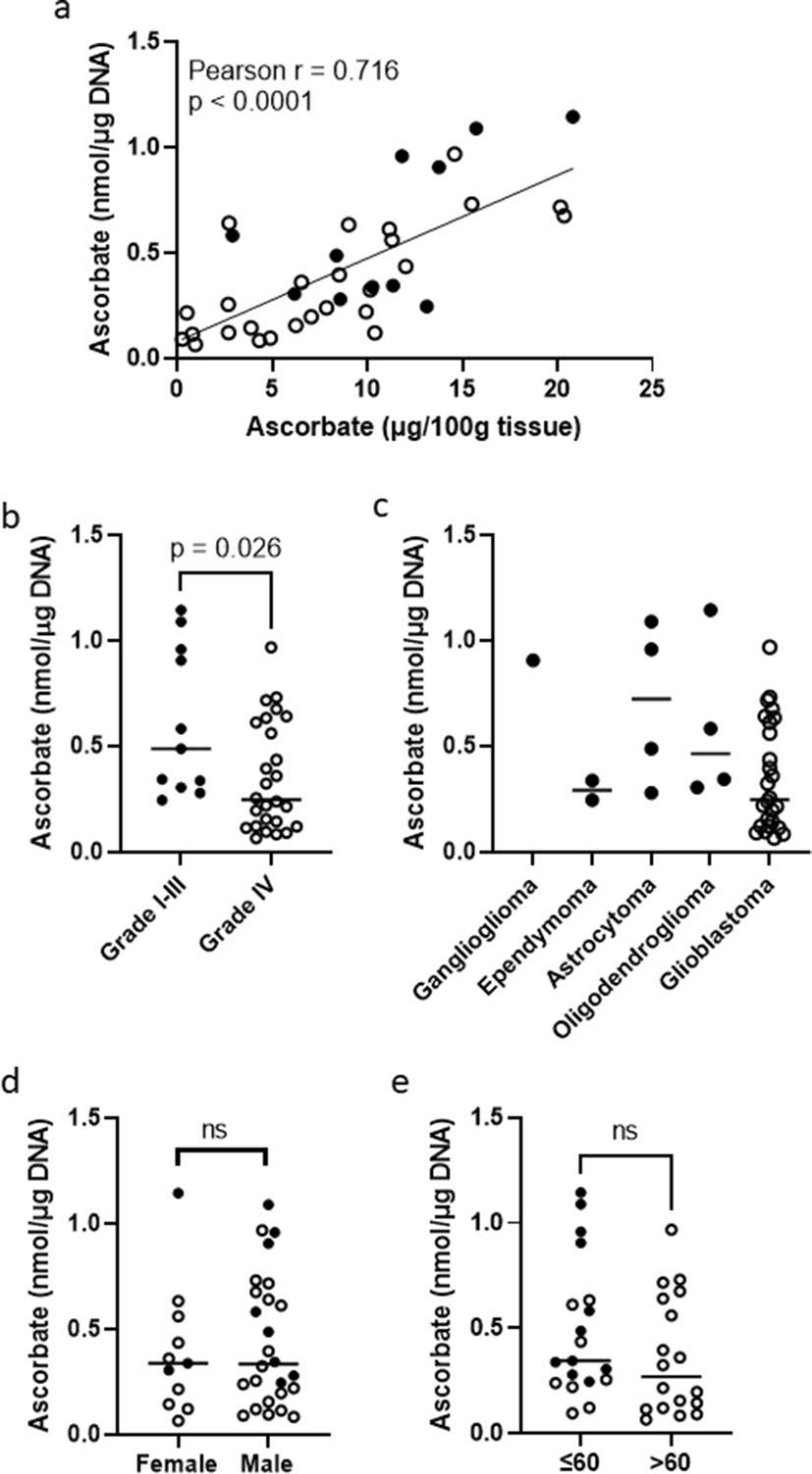
Ascorbate content of clinical glioma tissue from 37 patients. (**a**) Correlation between ascorbate content normalised to gDNA or normalised to tissue weight. Pearson correlation, significance indicated. (**b**) Ascorbate levels were higher in grade I-III (LGG, n = 11) than grade IV (HGG, n = 26) gliomas; unpaired two-tailed t-test. (**c**) Ascorbate levels varied across gliomas with differing subtypes, including ganglioglioma (n = 1), ependymoma (n = 2), astrocytoma (n = 4), oligodendroglioma (n = 4), and glioblastoma (n = 26). (**d**) Tumour ascorbate levels were similar in female (n = 11) and male (n = 28) patients, and (**e**) ascorbate levels did not differ by age (below (n = 19) or above (n = 18) 60 years), unpaired two-tailed t-tests. Filled circle WHO grade I–III, Open circle WHO grade IV; median is indicated by a horizontal line; ns, no significance.

Intracellular ascorbate levels for each glioma sample were calculated by normalising total ascorbate to either gDNA content or tissue weight (Fig. 1a). Ascorbate standardised to gDNA or tissue weight were strongly correlated (Pearson r = 0.716, *p* < 0.0001, Fig. 1a). Glioma ascorbate content averaged 0.428 ± 0.306 nmol ascorbate/µg DNA (median 0.338 nmol/μg DNA). In the duplicate astrocytoma samples, ascorbate levels were lower in the central (0.405 nmol/µg DNA) compared to peripheral (0.583 nmol/µg DNA) region of the sample (Supplementary Table 1). Location within the tumour mass was not recorded for most other samples. The recurrent oligodendroglioma had similar ascorbate levels in the initial (1.136 nmol/µg DNA) and recurrent sample (1.088 nmol/µg DNA).

Ascorbate content was significantly lower in HGG (mean 0.353 ± 0.258 nmol/µg DNA) compared to LGG tumours (mean 0.607 ± 0.349 nmol/µg DNA, *p* = 0.026; Fig. 1b). Tumour ascorbate levels varied between different histological subtypes (ganglioglioma, ependymoma, astrocytoma, oligodendroglioma and glioblastoma) (ANOVA *p* > 0.05; Fig. 1c), indicating that the higher ascorbate content of LGG tumours was not driven by a particular subtype. We also saw no difference in ascorbate content between female and male patients (p > 0.05; Fig. 1d), or between patients below or above 60 years of age (*p* > 0.05, Fig. 1e). There was no significant difference in ascorbate content when comparing *IDH1* wild type and *IDH1 R132H* tumours (*p* = 0.09, Supplementary Fig. 2A).

### Cytosine species in glioma

**Figure 2:**
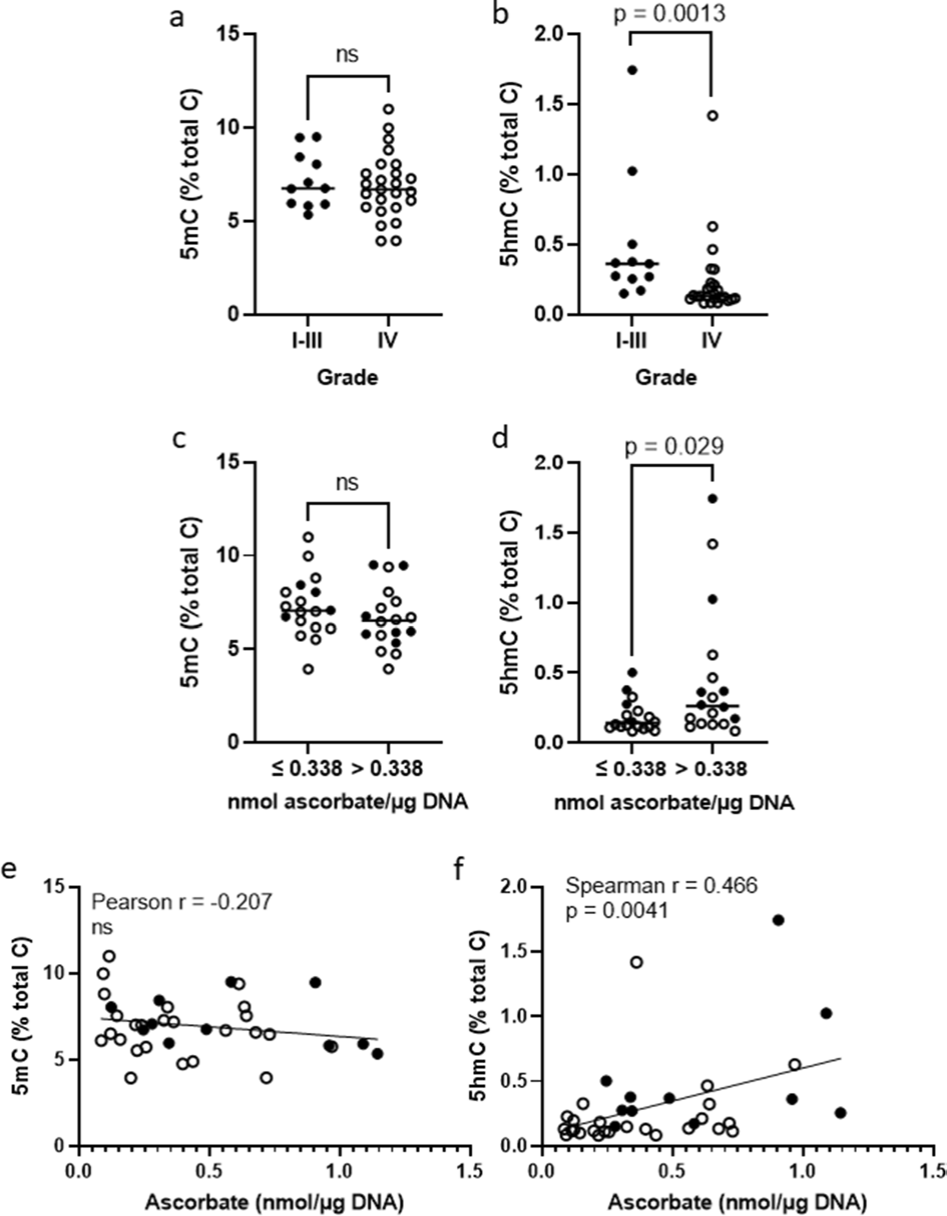
Relative levels of global cytosine species in clinical glioma samples according to tumour grade and ascorbate. Cytosine species include methylcytosine (5-mC) and hydroxymethylcytosine (5-hmC). Association between WHO grade I–III (n = 11) and grade IV (n = 25) tumours and (**a**) 5-mC and (**b**) 5-hmC levels. Association between below and above median ascorbate content (n = 18 each) and (**c**) 5-mC and (**d**) 5-hmC levels. (**e**) There was no association between 5-mC and ascorbate. (**f**) There was an association between 5-hmC and ascorbate. Individual samples (n = 36) are shown with the median as a horizontal line; Filled circle WHO grade I–III, Open circle WHO grade IV.

Global levels of all cytosine species, including methylated (5-mC) and hydroxymethylated (5-hmC) cytosine, were determined using mass spectrometry in glioma tissues (n = 36, one tumour did not provide sufficient DNA). Of the cytosine species, 6.97% were methylated and 0.32% were hydroxymethylated (Fig. 2). Hydroxymethylated cytosines are likely products, and a reflection, of TET enzyme activity, not TET protein levels^33^.

When cytosine species levels were compared between low- and high-grade gliomas, methylated cytosine proportions were similar between LGG and HGG (Fig. 2a), and glioma types (not shown). However, 5-hmC levels were significantly lower in HGG compared to LGG (0.236 ± 0.278% vs. 0.501 ± 0.476%, *p* = 0.0013, Fig. 2b), confirming previous reports^22^. There was no difference in the proportion of any of the cytosine species between *IDH1* wild type and mutant tumours (Supplementary Fig. 2B–D). Levels of 5-mC and 5-hmC were similar between the samples taken from initial and recurrent gliomas in the same patient, but showed some differences comparing the central and peripheral region of the same tumour (Supplementary Table 1).

### Association between ascorbate and global methylation status

The cohort was divided into below or above median ascorbate levels (0.338 nmol/μg DNA) to explore any associations between ascorbate and methylation status. No differences in 5-mC levels were observed between gliomas with below or above median ascorbate levels (7.27 ± 1.65% and 6.67 ± 1.62% for below and above median ascorbate, respectively, Fig. 2c). Remarkably, gliomas with above median ascorbate content showed significantly increased 5-hmC levels compared to below median ascorbate levels (0.19 ± 0.11% and 0.45 ± 0.47% for below and above median ascorbate, respectively, t test *p* = 0.029) (Fig. 2d). We saw no correlation between 5-mC levels and ascorbate content (Pearson r = − 0.207, Fig. 2e). In comparison, a solid association was evident between ascorbate and global levels of 5-hmC, in which increased ascorbate content was correlated with higher 5-hmC levels (Spearman r = 0.466, *p* = 0.004, Fig. 2f). These observations appeared to be independent of tumour grade (solid vs open symbols in Fig. 2c–f).

### Tumour-infiltrating macrophages

**Figure 3:**
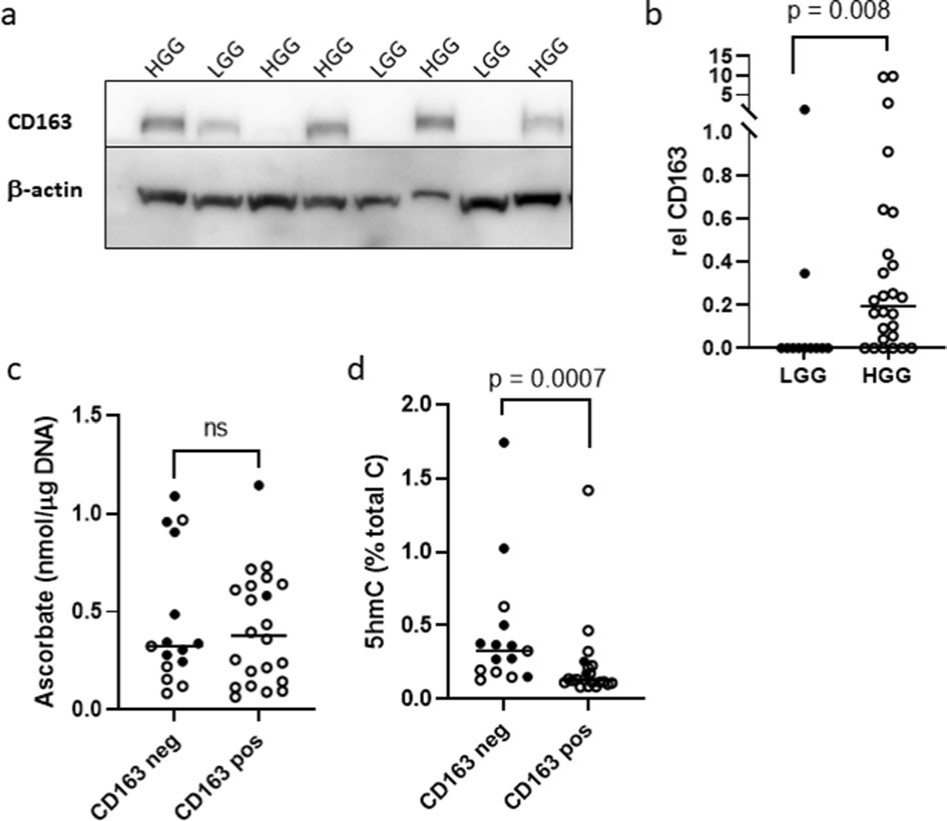
Infiltrating macrophages in glioma tumours. (**a**) Relative CD163 was assessed using Western blotting; a representative blot is shown. (**b**) High grade gliomas (HGG) had higher levels of CD163 than low grade gliomas (LGG), indicating a higher macrophage infiltrate. (**c**) Ascorbate content was similar between CD163 negative and positive tumours. (**d**) Hydroxymethylcytosine (5-hmC) was significantly lower in CD163 positive tumours, Mann Whitney test. Individual samples (n = 36) are shown with the median as a horizontal line; Filled circle WHO grade I–III, Open circle WHO grade IV; T, tumour; ns not significant.

Glioma tumours are heterogenous and contain numerous cell types besides cancer cells^34^, which may affect measurements. One potential contributor to cellularity and ascorbate content is the immune infiltrate. Cells of monocyte/macrophage lineage and activated microglia-positive macrophages can make up > 35% of tumour tissue^35^. We monitored CD163, which is exclusively expressed on these cells, by Western blot analysis, using anti-CD163 (full size blot Supplementary Fig. 3), to provide an indication of macrophage content of the tissue samples (Fig. 3a). Protein levels of CD163 were significantly higher in HGG compared to LGG (Mann–Whitney, p = 0.008, Fig. 3b), and more HGG had any staining compared to LGG (Fisher’s exact, *p* = 0.0023), indicating the presence of a higher immune infiltrate in the more aggressive glioblastomas, as described previously^35^. The percentage immune cells in the tissue could not be determined, as Western blot analysis was employed.

Brain ascorbate levels are among the highest in the body, with intracellular levels in neurons being estimated to be up to 10 mM, with levels in glial cells being around 1 mM^36^. However, leukocytes are also known to have high intracellular ascorbate levels (monocytes around 3 mM^37^), raising the possibility that the macrophage immune infiltrate may have contributed to the measured ascorbate levels. However, CD163 negative and CD163 positive tumours had similar ascorbate content (Fig. 3c), indicating that the macrophage ascorbate content did not drive tissue ascorbate levels. CD163 positive tumours had a significantly lower 5-hmC percentage than CD163 negative tumours (Mann–Whitney *p* = 0.0007, Fig. 3d), which may suggest that tumour cells contribute to the 5-hmC signal rather than macrophages. Most CD163 negative tumours were LGG. We therefore repeated the analyses for HGG only; ascorbate content was not different between CD163 positive/negative HGGs (*p* = 0.53), whereas CD163 positive HGGs tended to have lower 5-hmC levels than CD163 negative tumours (*p* = 0.059).

### MGMT promoter hypermethylation status in glioma samples

**Figure 4:**
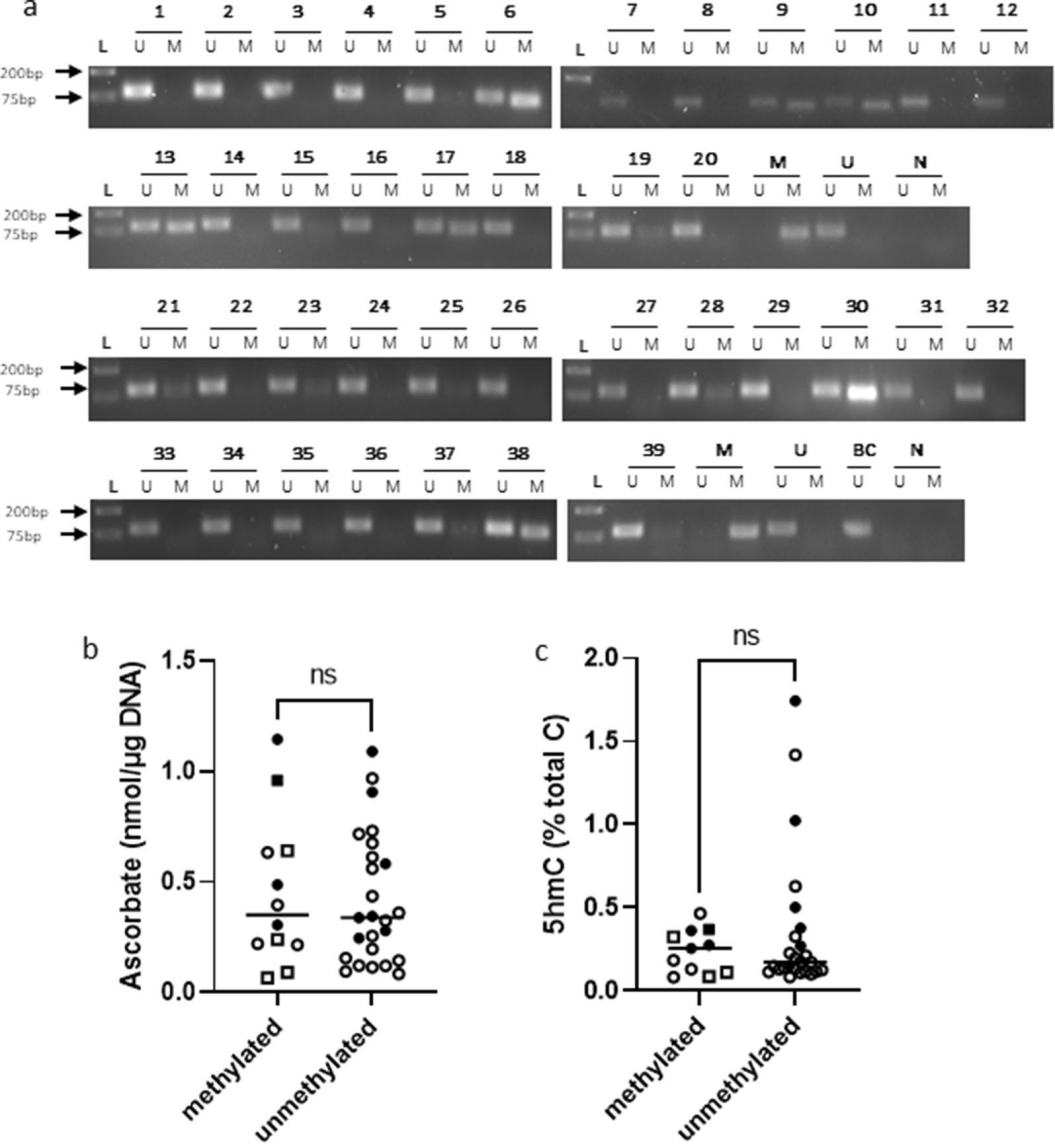
MGMT promoter hypermethylation status of clinical glioma samples. (**a**) Gel electrophoresis images of unmethylated (93 bp) and methylated (81 bp) MSP products for each glioma sample. The assay included an unmethylated (U), a methylated (M) positive control and a negative (N) control without DNA. Bisulfite conversion of all samples was performed simultaneously and the positive control reaction was visualised (BC). N = 39, including the two repeat samples (#24 and #36). (**b**) Association of ascorbate levels (nmol/µg/DNA) between gliomas with either unmethylated or methylated MGMT promoters. (**c**) Association of global 5-hmC (% of total cytosine) with unmethylated/methylated MGMT promoter. Filled circle WHO grade I-III, Open circle WHO grade IV, Square samples with apparent low methylation; median is shown as horizontal line, n = 38.

MGMT promoter hypermethylation status is used clinically to identify patients more likely to benefit from temozolomide treatment, with ‘methylation positive’ tumours more likely to respond as DNA repair is suppressed^17^. Methylation status of all tumours was assessed by bisulfite conversion of genomic DNA followed by methylation specific PCR (MSP). Hypermethylated promoters were represented by the presence of MSP product in both unmethylated and methylated reactions, whereas unmethylated promoters were evidenced by the presence of unmethylated product only and a lack of methylated MSP product. Twelve out of thirty-seven glioma samples were identified as having MGMT promoter methylation (32%; Fig. 4). Five of the twelve methylated samples had lower apparent levels of methylation (patients 19, 21, 23, 28, and 37; Fig. 4a; full size gel images are shown in Supplementary Fig. 4). Our data agreed with the clinical findings for all 3 tumours that had their status previously tested.

There was no difference in the proportion of MGMT promoter hypermethylated tumours between LGG (4/12 = 33%) and HGG (7/25 = 28%) (closed and open circles in Fig. 4b). Gliomas with unmethylated and methylated MGMT promoters had similar ascorbate levels (average ascorbate levels of 0.42 ± 0.30 nmol/µg/DNA and 0.45 ± 0.34 nmol/µg/DNA, respectively, t test *p* = 0.96; Fig. 4b), and this remained when gliomas with low and high apparent methylation levels were assessed separately (ANOVA *p* = 0.84; Supplementary Fig. 5). Between tumours with methylated or unmethylated MGMT promoter, there was no difference in global 5-hmC levels (Mann–Whitney *p* = 0.97, Fig. 4c), or 5-mC levels (t test, *p* = 0.92, not shown). This indicates that hypermethylation status of the MGMT promoter was not associated with global methylation or with ascorbate availability.

### Clinical outcomes

Patients were followed up for up to 19 years and survival was assessed using univariate analysis (Table 3). Patients with HGG had significantly shorter survival than those with LGG (HR 3.88, *p* = 0.009, Table 3), as expected from the fundamentally different pathophysiology of these tumours^2^. Patients with LGG had a median survival of 55 months, compared with 11 months for patients with HGG. Older age, male gender and *IDH1* wild type genotype were predictive of significantly poorer outcome (Table 3). Tumour ascorbate, cytosine species, MGMT promoter methylation and treatment were all not associated with survival in this cohort (Table 3), likely because survival differences by grade dominated any other relationship. We previously reported an association between longer survival and higher ascorbate in a cohort of patients with glioblastoma^32^, which included the patients with HGG analysed in this study, but no association was evident in this smaller cohort. Higher 5-hmC levels tended to associate with longer survival (HR 1.969, 95% CI 0.880–4.408, *p* = 0.099, Table 3), but this trend disappeared when adjusted for grade (HR = 1.323, 95% CI 0.562–3.125, *p* = 0.521).

**Table 3:** Univariate analysis of survival of patients with glioma in cohort.

Variable	Adverse factor	HR	Lower CI	Upper CI	p*
Grade	HGG	3.88	1.408	10.670	**0.009**
Age	Older	1.036	1.010	1.063	**0.006**
Gender	Male	2.698	1.224	5.947	**0.014**
IDH1	Wild type	3.323	1.104	9.996	**0.033**
Ascorbate	Low	1.129	0.534	2.389	0.750
Cytosine	High	1.132	0.529	2.421	0.749
5-mC	High	1.305	0.611	2.790	0.492
5-hmC	Low	1.969	0.880	4.408	0.099
MGMT	Methylated	1.438	0.624	3.314	0.393
Chemotherapy	No	1.506	0.700	3.239	0.295
Radiation	No	0.810	0.322	2.035	0.654
Chemoradiation	No	1.280	0.591	2.772	0.531

HR, hazard ratio; CI, 95% confidence interval.

*p-values were derived from the Cox regression model, statistical significance *p* < 0.05 in bold.

## Discussion

Our study is the first to measure ascorbate levels in clinical samples from both low-grade and high-grade gliomas, and to show that glioblastomas had significantly lower tumour ascorbate levels than lower-grade gliomas. We also demonstrated a strong positive correlation between tumour ascorbate levels and 5-hmC levels, supporting the notion of ascorbate as an essential cofactor for TET enzymes that are responsible for 5-hmC generation.

The difference in ascorbate content between low-grade and high-grade gliomas agrees with previous reports in endometrial, colorectal and breast cancer, where higher grade was associated with significantly lower tumour ascorbate levels^38–40^. In contrast, studies in renal cell carcinoma and thyroid cancer showed no association between grade and tumour ascorbate content^41,42^. Our current working hypothesis for an association between grade and ascorbate is based on ascorbate penetrance into tumour tissue. High grade gliomas are characterised by poor perfusion and lower microvessel density^43^, which, we propose, leads to reduced ascorbate supply to tumour cells^44^. In the glioma samples, we observed that HGG contained necrosis and reduced levels of microvessel proliferation, with both necrosis and microvessel proliferation being signs of hypoxia^43^. In mouse models, microvessel density and hypoxia (pimonidazole) were inversely related to ascorbate content^45^. Poor vascular supply is also closely related to hypoxia, which, in part, arises from reduced oxygen diffusion from sparse and ineffectual blood vessels^46^. An in vitro/mathematical model has described how oxygen and ascorbate diffusion through tumour tissue are similar, resulting in areas with both a loss in oxygen and ascorbate^44^, a two-fold setback for 2-OGDD enzymes.

Most LGG in our cohort were *IDH1 R132H* whereas only two HGG were *IDH1 R132H* mutants, as expected^1,2,7^. Those HGG harbouring the *IDH1* mutation (astrocytoma grade 4) have likely progressed from LGG, previously called secondary glioblastoma^1,2^. The IDH1 R132H enzyme exhibits gain-of-function activity, converting 2-OG into D-2-hydroxyglutarate (2-HG), an oncometabolite that competitively inhibits TET activity [reviewed in 26]. Our data showed a weak trend of *IDH1 R132H* samples containing less ascorbate. It is conceivable that reduced ascorbate combined with the oncometabolite 2-HG in HGG further compromise TET function, but larger numbers of *IDH1 R132H* mutant HGG are required to test this hypothesis.

One previous study had reported ascorbate content of astrocytoma tumours (0.309 ± 0.068 nmol/μg DNA, 9.4 ± 2.3 μg/100 mg tissue, n = 11)^31^, which is within error of our measurements in LGG (0.607 ± 0.349 nmol/µg DNA, 11.178 ± 4.831 µg/100 mg tissue, n = 11) and HGG tumours (0.353 ± 0.258 nmol/µg DNA, 7.866 ± 5.657 µg/100 mg tissue, n = 26). These variations are likely due to differences in sample processing, methodology and sample selection. Our previous study in glioblastoma contained overlapping samples with our HGG (n = 26^32^).

Our data has shown intriguing associations between global 5-hmC and both grade and ascorbate content. Early investigations of DNA methylation in glioma^14^, relied on bisulfite conversion techniques, which does not differentiate between 5-mC and 5-hmC. Distinction between 5-mC and 5-hmC was later made possible through the introduction of oxidative bisulfite chemistry^47^, and using this approach research has discovered 5-hmC-specific binding proteins that function in both DNA repair and transcriptional regulation^48,49^. Thus, in addition to being a DNA demethylation intermediate, 5-hmC is now believed to have its own unique role in the epigenetic regulation of mammalian cells. The role of ascorbate in controlling 5-hmC-specific functions is an avenue of further research.

Mutations in *TET*s have frequently been reported for hematopoietic malignancies, but loss-of-function mutations in *TET1/2* genes have only recently been reported for gliomas^12^. Loss of *TET1* via copy number variation was found to be common in *IDH1* wild type HGG, but not *IDH1* wild type tumours^12^. A study, in other cancers, indicated that global 5-hmC levels remain stable even following changes in TET protein abundance^33^. In contrast, a recent study showed that epigenetic downregulation of *TET3* in HGG reduced global 5-hmC levels^11^. We hypothesise that changes in 5-hmC in glioma may occur in response to variation in TET activity, due to ascorbate and oxygen availability, in addition to changes in *TET* expression.

Our data demonstrated no association between ascorbate and MGMT promoter methylation status. This observation is highly clinically relevant. MGMT promoter methylation reduces expression of the DNA repair enzyme which removes the alkylated guanine nucleotides generated by temozolomide therapy^16,17^. Hence, if ascorbate-supported TET activity was to reduce MGMT promoter methylation, this would adversely affect response to temozolomide treatment. Our findings are aligned with previous cell culture studies, which suggested that MGMT promoter methylation occurs independently from a global change of 5-hmC, as subcellular localisation of TET1 to the nucleus was associated with both elevated 5-hmC levels and maintenance of MGMT promoter hypermethylation^50^.

A limitation of this study is the relatively low number of clinical samples, specifically LGGs, but suitably processed frozen brain cancer samples are scarce and statistical tests provided confidence that the reported associations are genuine.

Another limitation is that our approach is unable to account for the considerable molecular heterogeneity of gliomas^7^; our methods rely on homogenized tumour tissue which consists not only of cancer cells, but also of varying proportions of other cell types and acellular extracellular matrix. We addressed this by (a) assessing the DNA content of the tissue and (b) measuring the relative content of CD163-positive macrophages. Ascorbate standardisation against DNA and tissue weight were closely correlated, indicating that non-cellular matter was not a major confounder for ascorbate analysis in this tumour tissue. Analysis of CD163 indicated that macrophage content did not drive ascorbate or 5-hmC levels.

Our previous study in glioblastoma (n = 37) showed an association between ascorbate content of HGGs and patient survival^32^, but this was not seen in our cohort containing both LGG and HGG, likely confounded by grade, and by low numbers, when LGG were excluded. High dose ascorbate has been suggested, and tried in individual patients, for treatment of glioblastoma^51–53^, but it is currently not known whether glioma tissue ascorbate levels can be manipulated by ascorbate supplementation. Ascorbate uptake into the brain is complex, as the blood brain barrier prevents ascorbate transport directly from the blood to the central nervous system^54^. Ascorbate instead enters the brain through the choroid plexus and diffusion through the cerebrospinal fluid where concentrations are above plasma levels^55^. Plasma and cerebrospinal fluid ascorbate levels in healthy volunteers are largely correlated^56^, but the relationship between plasma and brain tissue ascorbate has not been reported. Low glioma ascorbate levels are likely to reflect (a) poor functional tumour vasculature and/or (b) low ascorbate concentrations in the cerebrospinal fluid.

Hyper- and hypo-methylation events have been suggested as potential therapeutic targets for treating gliomas^57^. Our data from clinical glioma samples demonstrated a striking correlation between ascorbate content and 5-hmC levels. In high grade serous ovarian cancer, reduced 5-hmC is a hallmark of poor prognosis and survival, and resistance to platinum-based chemotherapy^58^. In cell culture models, sensitivity to chemotherapy was restored by increasing 5-hmC using TET2 overexpression. Similarly, in head neck squamous cell carcinoma, reduced expression of TET2 was associated with reduced 5-hmC levels, larger and more advanced tumours, and poorer prognosis^59^. Overexpression of TET2 increased 5-hmC levels and inhibited cell proliferation, invasion and migration in cell culture, and inhibited tumour growth in a xenograft model. Pharmacological ascorbate increased TET2 and 5-hmC levels, and decreased proliferation and migration, while increasing apoptosis in HNSCC cells^59^. This was also confirmed in bladder cancer, pancreatic ductal adenocarcinoma and leukaemia, where ascorbate supplementation in vitro increased 5-hmC levels^30,60–62^. Most advances have been made in myeloid cancers, where simple oral ascorbate supplementation of patients was able to increase the 5-hmC/5-mC ratio in circulating mononuclear myeloid cells^63^. Our data combined with these studies suggests that ascorbate may support TET1/2 function in gliomas and lead to increased 5-hmC, but intervention phase 0 trials are first required to assess whether glioma ascorbate content can be manipulated.

## Conclusion

Our data in clinical samples from both low- and high-grade gliomas have shown reduced ascorbate content in HGG compared to LGG, and a correlation between ascorbate and global 5-hmC levels. Importantly, methylation of the MGMT promoter showed no relationship with ascorbate. This supports the hypothesis that ascorbate supports TET1/2 activity, without changes to MGMT promoter methylation. These findings open the door to clinical intervention trials in patients with glioma in order to provide both mechanistic information and potential avenues for adjuvant ascorbate therapy.

## Materials and methods

### Patients and ethics

Glioma samples were gifted to He Taonga Tapu Cancer Society Tissue Bank (CSTB) Christchurch upon surgical removal of the tumour. All methods were performed in accordance with the relevant guidelines and regulations. Clinicopathological data, patient characteristics and follow-up data were collected from pathology reports and medical records.

### Patient samples

A total of 39 glioma samples from 37 patients, collected between 2003 and 2019, were analysed in this study. This cohort includes 26 glioblastoma samples that were also part of a related study on hypoxia, and where ascorbate levels and clinical follow-up have been published^32^. Two patients each provided two samples; one astrocytoma tumour was sampled twice during the same resection (central and periphery), and one patient provided two samples from recurrent oligodendroglioma tumours 3 months apart (following two previous resections over 6 years). The central astrocytoma sample was chosen as it contained ample DNA (indicating cellular matter) and to avoid contamination with unaffected brain. The initial oligodendroglioma sample was chosen to align better with the other samples from primary tumours. Only the central astrocytoma sample and the initial oligodendroglioma sample were included in figures, tables and statistical analyses; individual data for these duplicate samples is provided as supplementary material (Supplementary Table 1).

### Sample processing

Glioma samples were flash frozen in liquid nitrogen within 10–45 min of excision, reducing both warm and cold ischemia, and stored at − 80 °C. Frozen glioma samples (10–40 mg each) were homogenised by crushing into a fine powder in liquid nitrogen using mortar and pestle on dry ice. Powder was split into two portions for ascorbate measures and DNA extraction.

### Ascorbate analysis

Tissue ascorbate was measured as described previously^32^. Briefly, frozen glioma powder was resuspended in potassium phosphate buffer (10 mM, pH 7.4) (Sigma-Aldrich, St Louis, MO, USA). Protein was precipitated using 0.54 M perchloric acid (Sigma) containing 100 μM diethylene triamine penta-acetic acid (Sigma), the supernatant collected following centrifugation, and stored at − 80 °C until use. Total ascorbate was measured; any dehydroascorbate present was reduced to ascorbate by incubation with 32 mM tris(2-carboxyethyl)phosphine (Sigma) for 3 h on ice prior to analysing samples in duplicate on the Ultimate High Performance Liquid Chromatography unit (HPLC, Thermo Fisher Scientific, Waltham, MA, USA). Analysis was performed in reversed phase separation mode coupled to an electrochemical detector (Ultimate 3000 ECD-3000RS electrochemical detector and a Model 6011RS coulometric cell). The column oven was maintained at 30 °C and the autosampler at 4 °C. The samples (20 µL) were injected with the mobile phase (80 mM sodium acetate, 0.54 mM DTPA, 0.017% n-octylamine) running at a constant rate of 1.2 mL/min. A standard curve ranging from 1.25 to 40 µM ascorbate using sodium-L-ascorbate (Sigma) was made fresh for each run. Ascorbate content for the 26 glioblastoma samples has recently been published^32^.

### DNA extraction and preparation

Genomic DNA was purified from frozen glioma powder (~ 10 mg) using the PureLink Genomic DNA Kit (Thermo Fisher Scientific), following the manufacturer’s instructions. Total concentration of extracted genomic DNA was determined using Qubit dsDNA HS Assay (Thermo Fisher Scientific). Genomic DNA was subjected to either genomic digestion (for mass spectrometry) or bisulfite conversion (for Methylation Specific PCR (MSP)). Mass spectrometry was used to investigate global DNA methylation by quantifying three different cytosine species: cytosine, 5-methylcytosine (5-mC) and 5-hydroxymethylcytosine (5-hmC). Bisulfite conversion, which converts unmethylated, but not methylated, cytosine to uracil, followed by MSP, was used to determine methylation status of specific CpG islands in the MGMT promoter.

### Sample quality of glioma samples and DNA content

To evaluate sample integrity, gDNA and ascorbate content were assessed across the 16 years of sample storage. No significant change in DNA or ascorbate content was detected over this long sampling and storage period, providing confidence in sample quality (Supplementary Fig. 1A,B). DNA content of the clinical samples was assessed using Qubit which specifically measures double-stranded, genomic DNA (gDNA), and hence provides an indication of tumour cellularity, whereas tumour weight may not distinguish cells from non-cellular matter (lipid, fibrous material, extracellular matrix, etc.). gDNA content varied between samples, with an average of 0.265 ± 0.144 μg DNA/mg tissue, but showed no significant difference between LGG and HGG (Supplementary Fig. 1C).

### Global DNA methylation analysis by mass spectrometry

Purified genomic DNA (1 µg) was completely digested into single nucleosides using the Nucleoside Digestion Mix (New England BioLabs, Ipswich, MA, USA). Digestions were performed in 20 µL reaction volumes including Nucleoside Digestion Mix Reaction Buffer, Nucleoside Digestion Mix and purified genomic DNA, and incubated at 37 °C for 16 h to ensure complete digestion. Standards, containing known levels of 2′-deoxycytidine, 5-methyl-2′-deoxycytidine and 5-hydroxy-methyl-2′-deoxycytidine, and digested glioma DNA samples, were analysed using a 6500 QTrap mass spectrometer (Sciex, Framingham, MA, USA) coupled to an Infinity 1290 LC system (Agilent, Santa Clara, CA, USA). Standards and samples were stored on the autosampler tray at 5 °C. An Acclaim RSLC Polar Advantage II 120 Å column (150 × 2.1 mm, Thermo Fisher Scientific) was used for chromatographic separation using water (0.1% formic acid) as Solvent A and acetonitrile (0.1% formic acid) as Solvent B. A flow rate of 0.2 mL/min was used, with the column temperature set to 40 °C. The analytes eluted during the initial isocratic phase with 100% Solvent A over 3.5 min. The column was then flushed with 5% Solvent A and 95% Solvent B for 2.5 min, and then re-equilibrated at initial conditions for 5 min. Data were analysed using Analyst 1.7.1 (Sciex). All three species were quantified by fragmenting the singly-charged parent ion [M + H]^+^, monitoring the fragment ion resulting from the loss of the deoxyribose sugar in positive-ion mode (Supplementary Table 2), and then measuring the area under the curve of the resulting peak (Fit: Linear, Weighting: None, Regression Parameter: Area). The sodium adduct ion [M + Na]^+^ of each species was also detected, but not quantified and used only as confirmation. Global levels of each of the cytosine species, cytosine (unmethylated), 5-methyl-cytosine (5-mC), and 5-hydroxymethyl-cytosine (5-hmC), were determined as a percentage of the total cytosine species levels (sum of cytosine, 5-mC and 5-hmC, detected as their corresponding nucleosides, 2’-deoxycytidine, 5-methyl-2’-deoxycytidine and 5-hydroxy-methyl-2’-deoxycytidine, respectively).

### Methylation specific PCR (MSP)

Purified genomic DNA (200 ng) was subjected to bisulfite conversion using the EZ DNA Methylation Kit (Zymo Research), following manufactures instructions. MSP was carried out using KAPA LongRange HotStart DNA Polymerase (Sigma-Aldrich) and included 1X KAPA LongRange Buffer, 6.7 mM of MgCl_2_, 1.25 mM KAPA dNTP mix, 300 ng of each primer, 1.25U of KAPA LongRange HotStart DNA Polymerase, 50 ng genomic DNA, in ultra-pure water. For each DNA sample, two distinct PCR reactions were performed using the MGMT primer pairs outlined in Supplementary Table 3, as previously described^20^. Briefly, the MGMT promoter contains 98 CpG sites, which have been assigned consecutive numbers (5′ to 3′) starting with CpG1 (− 452 bp from transcriptional start site) through to CpG98 (+ 308 bp). CpGs 73–90 play a critical role in the transcriptional control of MGMT expression^64,65^, and were thus targeted for our MSP approach (9 CpGs in total). Each primer pair was specific to either methylated or unmethylated DNA sequences. Positive control DNA was the EpiTect PCR Control DNA set (Qiagen, Hilden, Germany), which included completely unmethylated human genomic DNA, completely unmethylated bisulfite converted human genomic DNA, and completely methylated bisulfite converted human genomic DNA. Unmethylated human genomic DNA was subjected to bisulfite conversion in parallel to the genomic DNA extracted from patient tissue. Negative controls without DNA were performed for each of the methylated and unmethylated primer reactions. Thermal cycling conditions, performed on the Mastercycler pro PCR system (Eppendorf, Hamburg, Germany), included an initial denaturation step at 95 °C for 5 min, 35 cycles of denaturation (95 °C, 30 s), annealing (59 °C, 30 s) and extension (72 °C, 30 s), and a final extension at 72 °C for 4 min. PCR products were visualised on 2% agarose gels (agarose, TAE buffer and SYBR Safe DNA Gel stain (Life Technologies, Carlsbad, CA, USA)).

### *IDH1* mutation status

Purified genomic DNA (40 ng) was used for PCR to amplify a 496 bp region of DNA encoding exon 4 of *IDH1* (primer sequences 5′-AATGAGCTCTATATGCCATCACTG-3′ (forward) and 5′-TTCATACCTTGCTTAATGGGTGT-3′ (reverse)), as described before^32^. *IDH1* status for the 26 glioblastoma samples has previously been published^32^.

### Western blot analysis

Frozen tissue powder was homogenised in RIPA buffer (50 mM Tris (pH 8), 150 mM NaCl, 1% IGEPAL, 0.5% sodium deoxycholate. 0.1%, 0.5% sodium dodecyle-sulfate, with Complete Protease Inhibitor Cocktail) (Sigma-Aldrich). Prior to loading onto a 4–12% gradient Bis–Tris Plus SDS gel (Life Technologies, Carlsbad, USA), samples were mixed with sample buffer (60 mM Tris pH 4.8, 2% sodium dodecyle sulfate, 20% glycerol, 0.01% bromophenol blue, 0.1 M dithiothreitol) and boiled. Loading was standardised to 0.4 µg DNA per lane. Proteins were transferred to 0.45 µm polyvinylidene difluoride membrane, blocked in 5% skim milk, incubated overnight at 4 °C with primary antibodies (anti-CD163, 1:1000, ab182422 Abcam; anti-*β*-actin, 1:5000, Sigma A5441), followed by incubation with horseradish peroxidase-conjugated secondary antibodies (anti-rabbit, anti-goat or anti-mouse). Immuno-bands were visualised with ECL Select Western Blotting Detection Reagent (RPN2235, Cytiya) and semi-quantified using the NineAlliance imaging system (Uvitec, Cambridge, UK).

### Statistical analysis

Data were analysed with GraphPad Prism (Version 5.03). The Schapiro-Wilks normality test was used to determine the distribution of each set of data. Associations between ascorbate and clinicopathological characteristics were assessed using unpaired t-tests, Mann–Whitney tests or ANOVA, for parametric or non-parametric data, respectively. Binary data was assessed using Chi-square or Fisher’s exact test, as appropriate. Pearson or Spearman’s correlations were used to investigate relationships between ascorbate normalised by DNA or by tissue weight content, and between ascorbate and global methylation levels, for parametric or non-parametric data, respectively. Hazard ratios were calculated by univariate and multivariate Cox proportional hazard modelling for clinicopathological and biochemical data using overall survival data.

### Ethical approval

Ethical approval for this study was granted by the University of Otago Ethics Committee (H19/163) and samples were approved for use by the Canterbury Tissue Bank Board (2001DPVR). Donors gave informed written consent for the use for research of their samples and access to medical records.

## Supplementary Information

Supplementary Information 1.

Supplementary Information 2.

## Data Availability

All data generated or analysed during this study are included in this published article (and its Supplementary Information files).
