# Supplementary material for: Ascorbate content of clinical glioma tissues is related to tumour grade and to global levels of 5-hydroxymethyl cytosine
Source: Sci Rep. 2022 Sep 1;12:14845. doi: 10.1038/s41598-022-19032-8 (PMC9436949; doi:10.1038/s41598-022-19032-8)
Supplement: Supplementary file 1 — Supplementary Information 1. [file 41598_2022_19032_MOESM1_ESM.pdf]

Ascorbate content of clinical glioma tissues is related to tumour grade and to global levels of 5-hydroxymethyl cytosine

Rebekah LI Crake, Eleanor R Burgess, George AR Wiggins, Nicholas J Magon, Andrew B Das, Margreet CM Vissers, Helen R Morrin, Janice A Royds, Tania L Slatter, Bridget A Robinson, Elisabeth Phillips, Gabi U Dachs

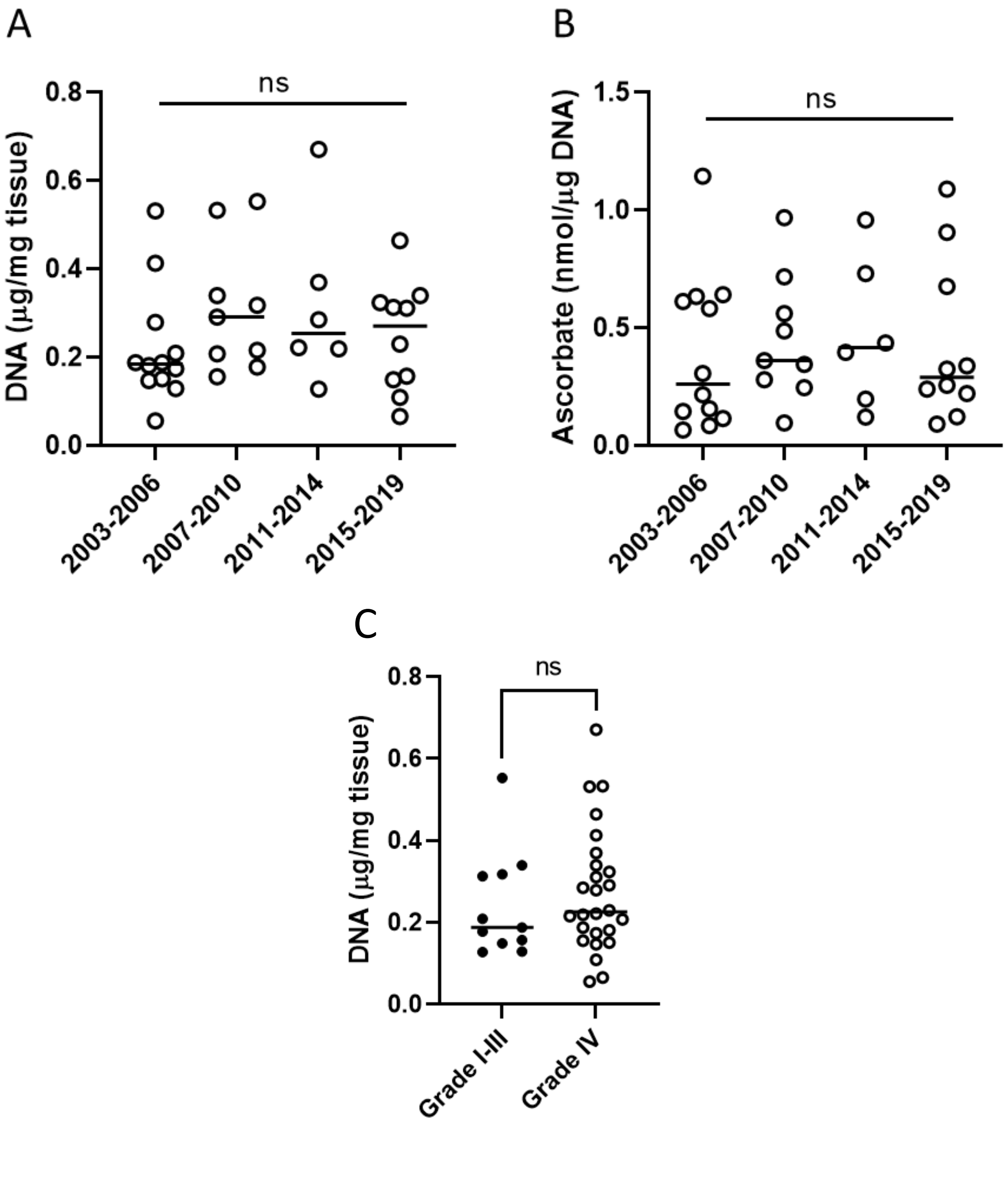

Supplementary Figure 1. Quality and integrity of glioma samples.

(A) Genomic DNA and (B) ascorbate content were assessed across 16 years of frozen storage of samples in the tissue bank. No significant change in DNA or ascorbate content was detected over this long sampling and storage period. (C) Genomic DNA (gDNA) content of clinical glioma specimens, separated into low (grade I-III) and high grade (grade IV). Median is indicated by a horizontal line, n=37, ANOVA, ns, not significant.

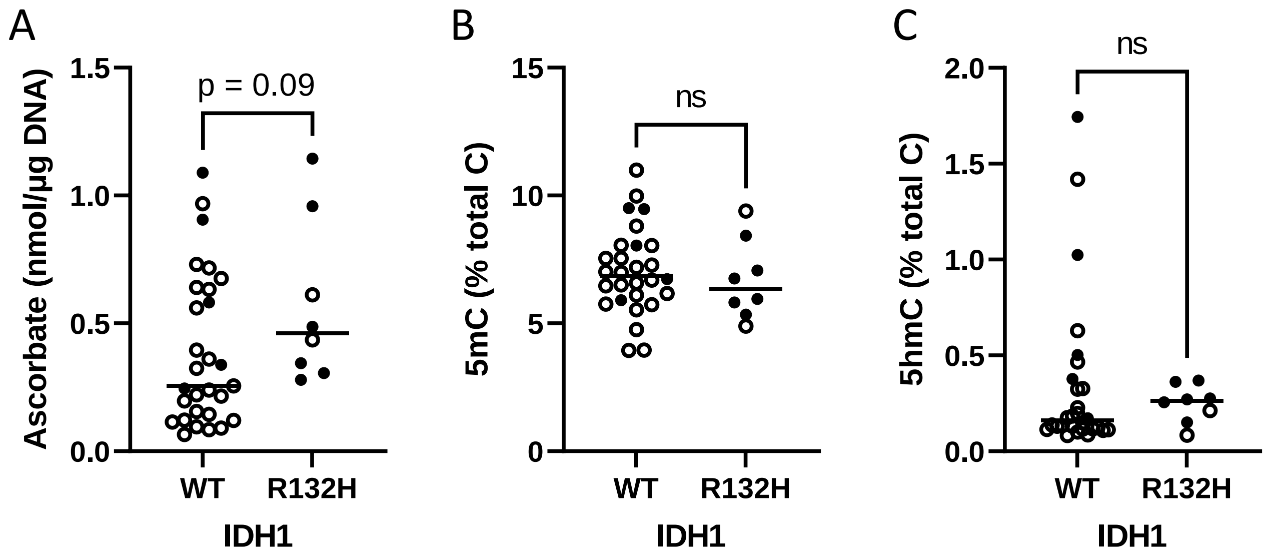

Supplementary Figure 2. Ascorbate content and global methylation status according to *IDH1* mutation status.

(A) There was no significant difference in ascorbate content when comparing *IDH1* wild type and *IDH1* R132H gliomas. (B, C) There was no difference in the proportion of 5-methylcytidine (5-mC) or 5-hydroxymethylcytidine (5-hmC) between *IDH1* wild type and mutant tumours. Median is indicated by a horizontal line; n=37, n= 29 *IDH1* WT, n=8 *IDH1* R132H mutant tumours; unpaired two-tailed t-tests, ● WHO grade I-III, ○ WHO grade IV; ns, no significance.

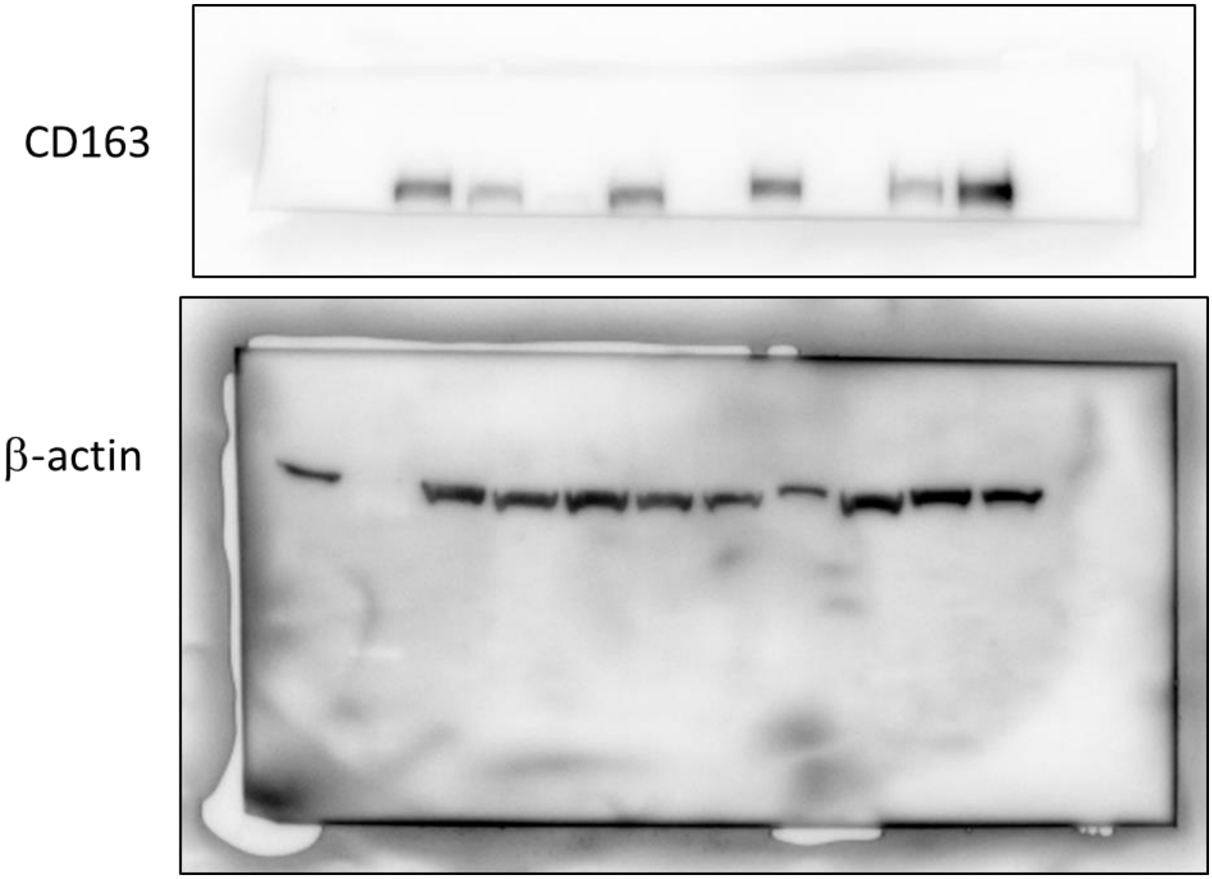

Supplementary Figure 3. Full size Western blots showing CD163 and β-actin staining.

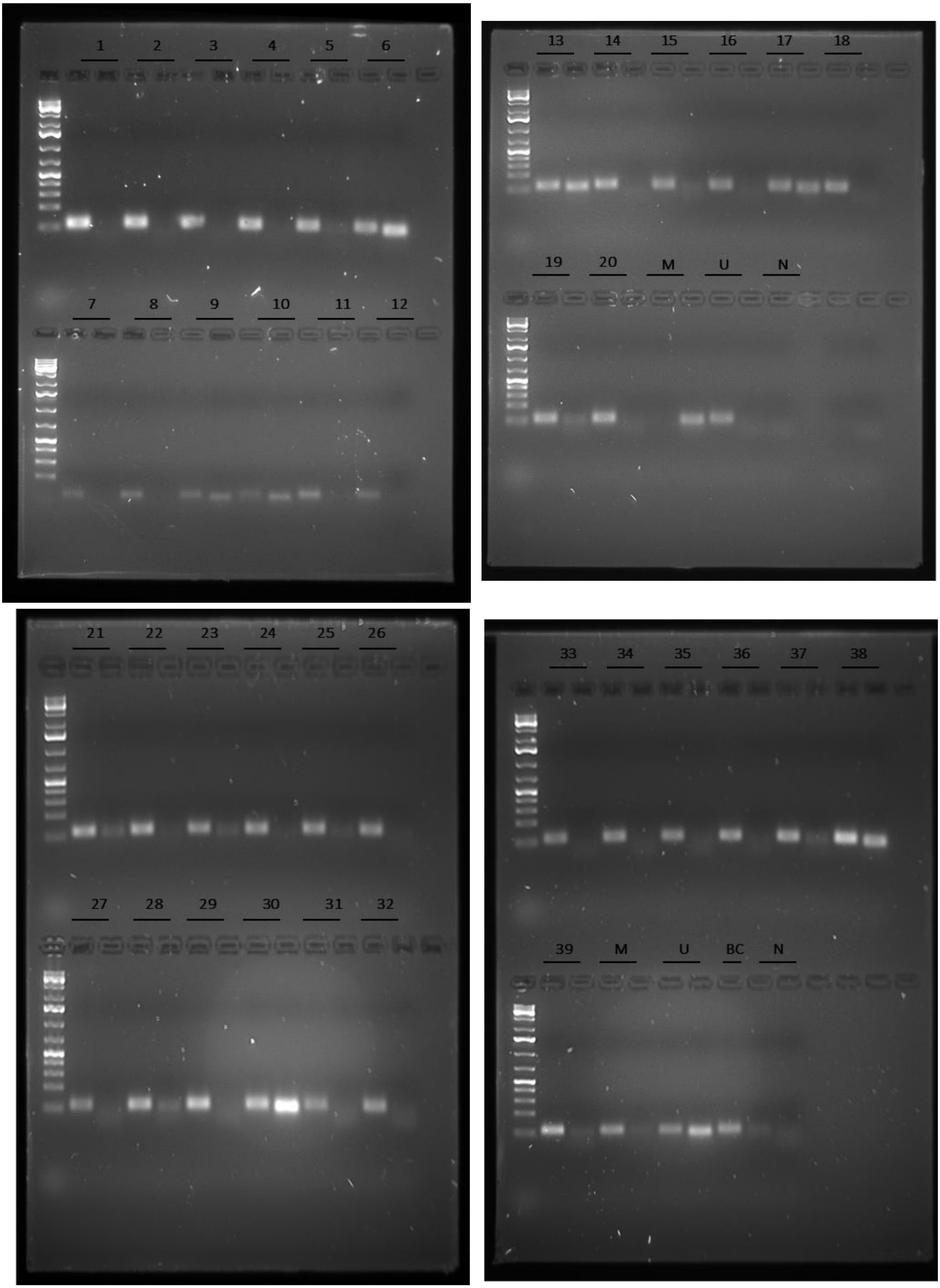

Supplementary Figure 4. Full size gel electrophoresis images of MSP products.

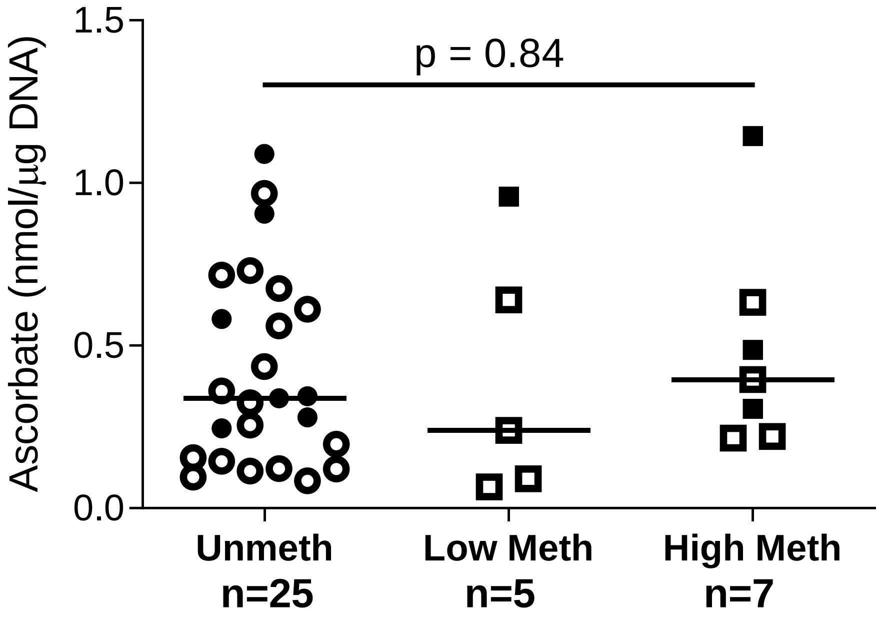

Supplementary Figure 5. Ascorbate content of glioma tumours according to MGMT promoter methylation status.

There was no significant difference in ascorbate content between gliomas that had unmethylated, low methylated or high methylated MGMT promoter according to methylation-specific PCR. Median is indicated by a horizontal line; n=37; ANOVA, ns, no significance; ●■ WHO grade I-III, ○□ WHO grade IV ;. ■□ methylated MGMT promoter.
