# Supplementary material for: Ascorbate content of clinical glioma tissues is related to tumour grade and to global levels of 5-hydroxymethyl cytosine
Source: Sci Rep. 2022 Sep 1;12:14845. doi: 10.1038/s41598-022-19032-8 (PMC9436949; doi:10.1038/s41598-022-19032-8)
Supplement: Supplementary file 2 — Supplementary Information 2. [file 41598_2022_19032_MOESM2_ESM.pdf]

Ascorbate content of clinical glioma tissues is related to tumour grade and to global levels of 5-hydroxymethyl cytosine

Rebekah LI Crake, Eleanor R Burgess, George AR Wiggins, Nicholas J Magon, Andrew B Das, Margreet CM Vissers, Helen R Morrin, Janice A Royds, Tania L Slatter, Bridget A Robinson, Elisabeth Phillips, Gabi U Dachs

Supplementary Tables Crake et al

Supplementary Table 1: Individual data for duplicate glioma samples.

	Patient A		Patient B	
	*Initial sample	Recurrent sample	Peripheral sample	*Central sample
**WHO grade**	3	3	3	3
**Glioma type**	Oligodendro glioma	Oligodendro glioma	Astrocytoma	Astrocytoma
**Specimen position**	Temporal lobe right	Temporal lobe right	Temporal lobe right	Temporal lobe right
**DNA content (mg/mg tissue)**	0.088	0.149	0.129	0.266
**Ascorbate (nmol/μg DNA)**	1.136	1.088	0.583	0.405
**IDH1 R132H Status**	Wild type	Wild type	Wild type	Wild type
**MGMT promoter hypermethylation**	unmethylated	unmethylated	unmethylated	unmethylated
**Cytosine (% of total)**	93.148	93.072	90.320	94.295
**Methylcytosine (% of total)**	5.797	5.904	9.507	5.473
**Hydroxymethyl-cytosine (% of total)**	1.055	1.024	0.173	0.232

*samples used in analyses

Supplementary Table 2. The m/z values for the singly-charged parent and fragment ions and the optimised parameters that were used to quantify each analyte in LC-MS/MS experiments.

Analyte	Parent (m/z)	Fragment (m/z)	DP	EP	CE	CXP
Deoxycytidine (H^+)	228.10	112.05	45	7	15	16
5-Methyl-deoxycytidine (H^+)	242.11	126.07	45	7	13	16
Hydroxymethyl-deoxycytidine (H^+)	258.11	142.06	50	7	11	16
Deoxycytidine (Na^+)	250.08	134.03	76	7	21	18
5-Methyl-deoxycytidine (Na^+)	264.10	148.05	76	7	21	18
Hydroxymethyl-deoxycytidine (Na^+)	280.09	164.04	76	7	21	18

DP (declustering potential), EP (entrance potential), CE (collision energy), CXP (cell exit potential).

Supplementary Table 3. PCR primer pairs.

MGMT MSP Target	Sequence (5' to 3')	Tm (°C)	Amplicon Length (bp)
Methylated Forward	TTT CGA CGT TCG TAG GTT TTC GC	57.9	81
Methylated Reverse	GCA CTC TTC CGA AAA CGA AAC G	56.9	
Unmethylated Forward	TTT GTG TTT TGA TGT TTG TAG GTT TTT GT	55.2	93
Unmethylated Reverse	AAC TCC ACA CTC TTC CAA AAA CAA AAC A	57.6	
